# Controllable Synthesis of Monodisperse Er^3+^-Doped Lanthanide Oxyfluorides Nanocrystals with Intense Mid-Infrared Emission

**DOI:** 10.1038/srep35348

**Published:** 2016-10-17

**Authors:** Huilin He, Qiang Liu, Dandan Yang, Qiwen Pan, Jianrong Qiu, Guoping Dong

**Affiliations:** 1 State Key Laboratory of Luminescent Materials and Devices and Guangdong Provincial Key Laboratory of Fiber Laser Materials and Applied Techniques, School of Materials Science and Engineering, South China University of Technology, Guangzhou 510640, China

## Abstract

Monodisperse lanthanide oxyfluorides LnOF (Ln = Gd, Y) with mid-infrared emissions were controllably synthesized via a mild co-precipitation route and a subsequent heat-treatment. The detailed composition and morphology were characterized by X-ray diffraction (XRD), scanning electron microscope (SEM) and high resolution transmission electron microscopy (HRTEM). The results showed that monodisperse GdOF:Er^3+^ were nano-riced shape with length about 350 nm and width about 120 nm, while the quasi-spherical YOF:Er^3+^ were uniform nanocrystals with an average size around 100 nm. The influence of calcination temperature on the size and phase transition of LnOF nanocrystals was also investigated. The photoluminescence (PL) spectra indicated that the 2.7 μm emission of Er^3+^ had achieved in both GdOF and YOF nanocrystals, which were calcined at different temperatures. In addition, the decay time of both ^4^*I*_13/2_ and ^4^*I*_13/2_ energy levels corresponding to Er^3+^ in YOF nanocrystals were also studied in detail. The results suggested that both rice-shaped GdOF nanocrystals and YOF nanocrystals could provide suitable candidate materials for nanocrystals-glass composites, which could be a step forward to the realization of mid-infrared laser materials.

In the past few years, the solid state lasers operating in the mid-infrared wavelength region (2–5 μm) have been widely investigated. Among them, the 2.7 μm mid-infrared emission of erbium ions (Er^3+^) has recently gained considerable attention due to its potential application in various areas, such as laser microsurgery, remote sensing, military counter-measures and environmental monitoring^1^,^2^,^3^,^4^,^5^. So far, Er^3+^ ions fluorescence at 2.7 μm, arising from ^4^*I*_11/2_ to ^4^*I*_13/2_ transition, has been realized in various glasses^6^,^7^,^8^,^9^,^10^, glass ceramics^11^,^12^ and single-crystals^13^,^14^,^15^,^16^. Moreover, the mid-infrared micro/nanocrystal-glass composites have been proposed to fabricate composite optical fiber for the realization of mid-infrared fiber lasers. Previously, the 2.7 μm emissions of Er^3+^ ions were observed in the transparent Er^3+^: CaF_2_-fluorophosphate glass microcomposite^17^ and yttrium aluminum garnet (Y_3_Al_5_O_12_) nanocrystals-tellurate glass composites^18^. However, the realization of efficient mid-infrared emission from Er^3+^ doped micro/nanocrystals has become one of the bottlenecks. The Er^3+^ (^4^*I*_11/2_ → ^4^*I*_13/2_ transition) is susceptible to the phonon vibration of host materials. In addition, higher phonon energy is favorable to the rate of nonradiative transitions, causing a detrimental effect on the upper-level to lower-level lifetime. On the other hand, a main factor affecting Er^3+^:2.7 μm fluorescence is the absorption of H_2_O around 3 μm, which corresponds to the stretching vibration of free OH^−^ groups and will quench the Er^3+^:2.7 μm fluorescence^7^,^11^,^14^,^15^,^16^,^17^. Therefore, in order to achieve efficient and intense 2.7 μm emission of Er^3+^ ions in the micro/nanocrystals for transparent glass composites, many stringent requirements regarding their host materials have been proposed. These requirements include low phonon energy, good thermal and chemical stability and the matched refractive index^7^,^11^,^14^,^15^,^16^,^17^. In addition, suitable size and morphology of Er^3+^ doped micro/nanocrystals are also important^18^.

Lanthanide oxyfluorides (LnOF) nanocrystals have attracted increasing attention since they are excellent host materials for efficient up/down conversion luminescence. Therefore, they can be widely used in the optoelectronic devices, solar cell, bioimaging, biodetection and photocatalysis^19^,^20^,^21^,^22^. LnOF possesses the advantages of both fluorides and oxides, i.e. low phonon energy, high iconicity, good optical transparency, good chemical and thermal stability. These advantages meet the glass composite requirements for efficient and intense emission and suggest that LnOF is an ideal host material candidate for mid-infrared emission. To the best of our knowledge, most studies on lanthanide ions doped LnOF nanocrystals focused on the visible and near-infrared emission properties. Few works have reported mid-infrared properties achieved in the GdOF host matrix, letting alone systematic investigation on the 2.7 μm emission realized in the LnOF (Ln = Gd, Y) nanocrystals. Therefore, as a step forward to achieve transparent mid-infrared nanocrystals-glass composites, the preparation of nanocrystals with suitable morphology and effective mid-infrared emission is still an intractable issue.

Herein, we report for the first time the realization of 2.7 μm emissions of Er^3+^ ions in the GdOF and YOF nanocrystals. We prepared the nanoscale Er^3+^-doped GdOF and YOF samples at different temperatures via a simple co-precipitation route and sequent heat-treatment. During the calcination process, the morphologies and phase transformation of precursor were studied in detail. The luminescence characteristics of Er^3+^-doped GdOF and YOF nanocrystals, which sintered at different temperatures, were studied. Moreover, the ^4^*I*_11/2_ and ^4^*I*_13/2_ levels lifetime of Er^3+^ ions in YOF nanocrystals were investigated. Based on experimental results we further discuss the potential of Er^3+^-doped GdOF and YOF as the eligible candidates for nanocrystals-glass composites.

## Results

### Controllable synthesis and mid-infrared properties of Er^3+^-doped GdOF nanocrystals

**Figure 1:**
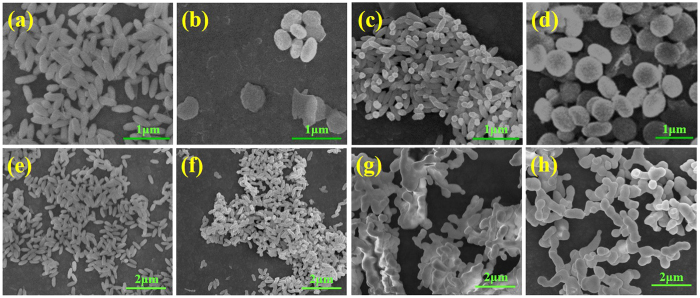
(**a–d**) SEM images of 0.05 mol Er^3+^ doped GdOF precursor: pH = 2, 33 mol urea, (**a**) GdOF-Er-a (1 mmol KF solution), (**b**) GdOF-Er-b (1 mmol KF solid powder), (**c**) GdOF-Er-c (1.2 mmol KF solution), (**d**) GdOF-Er-d (1.2 mmol KF solid powder). (**e–h**) SEM images of 0.05 mol Er^3+^ doped GdOF precursor sintered at different temperatures for 3 h: (**a**) 0 °C, (**b**) 650 °C, (**c**) 800 °C, (**d**) 1000 °C.

Developed from the conventional method reported by Lin’s group^20^, some changes had been made to synthesize the oxyfluoride (LnOF) precursor nanocrystals via a mild water-bath method. The detailed experimental parameters for different precursors corresponding to GdOF:0.05Er are listed in Supplementary Table 1 as GdOF- Er-(a-d), respectively (GdOF-Er-(a-d) are firstly discussed in this section). As the pH = 2 and the addition of 33 mmol urea, the state and amount of KF are variable. During experiments, the amount of urea (33 mmol) and the pH value (pH = 2) of solution remained constants. But the state and amount of KF were changed. Fig. 1(a–d) display the SEM images of 0.05 mol Er^3+^ doped GdOF precursor samples. Overall, the precursors present a morphology of nanoscale rice-like as the addition of KF solution, but it turns out to be a shape of sub-micron oblate spheroids when the KF solid powder was introduced. Fig. 1(a) is the SEM image of the precursor sample (120 nm in width, 400 nm in length) when 1 mmol KF was added into solution. The distribution of the rice-shaped nanocrystals is uniform. As an equivalent amount of solid KF was introduced, the sample presented a morphology of sub-micron oblate spheroids and several nano-sheets (Fig. 1(b)). With an introduction of 1.2 mmol KF solution, Fig. 1(c) exhibits the precursors a morphology of nano-sized rice with smaller size. Additionally, with 1.2 mmol solid KF as a contrast, Fig. 1(d) also displays some sub-micron oblate spheroids. It should be noted from the above results that both the state and dosage of KF play an important role in the morphology and size distribution of the GdOF:0.05Er precursors^23^. Since the urea is more easily hydrolyzed in the heavily acidic conditions (pH = 2), and the solid KF may hinder the nucleation process and decrease the nucleation rate, resulting in an isotropic growth and larger size in products. In addition, the GdOF**-**Er-a precursor sample has chosen to be investigated as a better choice.

**Figure 2:**
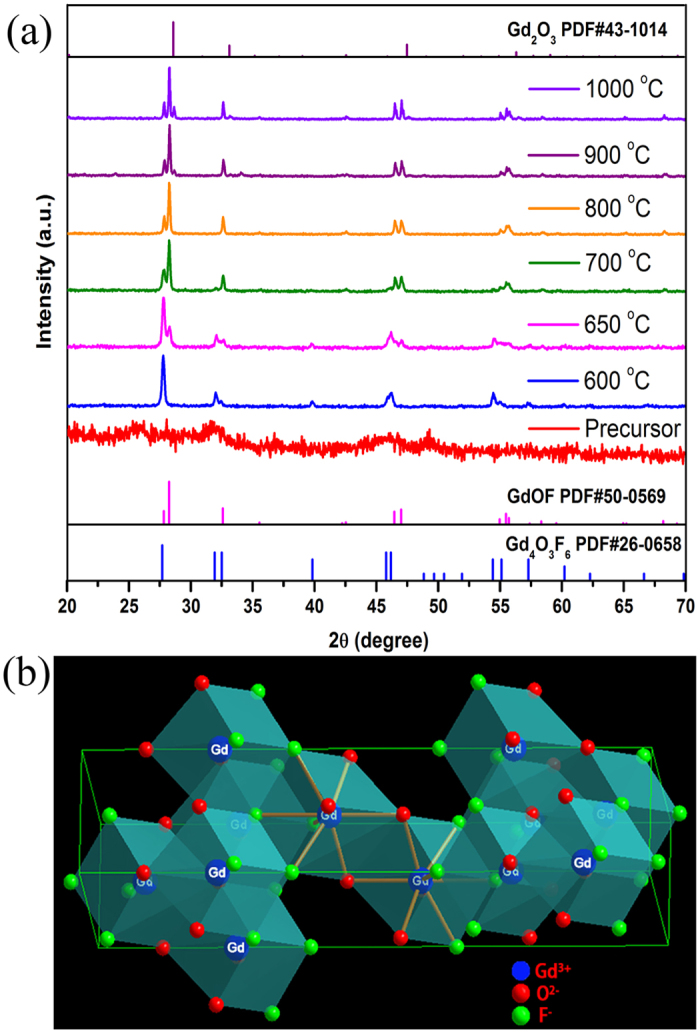
(**a**) XRD patterns of 0.05 mol Er^3+^ doped GdOF precursor sintered at different temperatures for 3 h. (**b**) Crystal structure of rhombohedral GdOF.

Based on the results shown in Fig. 1(a–d), the GdOF**-**Er-a precursor samples sintered at different temperatures have been further prepared. Fig. 2(a) shows the XRD patterns of as-prepared 0.05 mol Er^3+^ doped GdOF precursor sintered in the air at different temperatures. It can be clearly seen from Fig. 2(a) that the precursor sample exhibits non-obvious diffraction peaks, which indicates that the precursor is amorphous. As the temperature raised to 600 °C, clear diffraction peaks appeared and could be assigned to the pure Gd_4_O_3_F_6_ phase (a = b = 5.579 Å, c = 5.499 Å). And as the calcination temperature was 650 °C, the diffraction peaks show that rhombohedral phase of GdOF (a = b = 3.8648 Å, c = 19.258 Å) appears and Gd_4_O_3_F_6_ phase still exists, which means the occurrence of a crystalline phase transformation from Gd_4_O_3_F_6_. When the temperature went up to 700–800 °C, the diffraction peaks can be assigned to the pure rhombohedral phase of GdOF. However, with a further increase of temperature to 900–1000 °C, a few diffraction peaks corresponding to impurity phase of cubic Gd_2_O_3_ (a = b = c = 10.813 Å) generate in the patterns, coupled with the main GdOF rhombohedral phase. The XRD results show three period of phase transformation from Gd_4_O_3_F_6_ phase to rhombohedral GdOF, and then to cubic Gd_2_O_3_ with a rising temperature due to more oxygen introduced into the products during the calcination process. Moreover, with the increase of calcination temperature, the crystallinity becomes better, and the diffraction peaks become narrower, which indicates the size of products increase as elevating the temperature. Additionally, Fig. 2(b) displays the crystal structure of rhombohedral GdOF (CN = 8), the desired product, with the R-3m (146) space group, which shows the Gd cation sites are coordinated by four oxygen and four fluorine ions with a C_3v_ site symmetry^24^,^25^.

To shed light on the phase transformation of precursor in the heat-treatment process, the TG-DSC curves of the GdOF**-**Er-a precursor sample are presented in Supplementary Fig. S1. The DSC curve exhibits three phases weight loss. The first mass loss before 260 °C is ascribed to the loss of residual water and organic components, accompanying with an endothermic peak around 166.4 °C during this process. In addition, the endothermic peaks around 485.9 °C appears together with a great mass loss from 260 °C to 500 °C, which might be due to decomposition of the precursors and the formation the oxyfluoride (Gd_4_O_3_F_6_) crystalline phase, combined with the results in Fig. 2(a)^26^. It should be noted that the endothermic peaks around 682.7 °C, meanwhile, with a slight weight loss, might be ascribed to the phase transition from Gd_4_O_3_F_6_ to rhombohedral GdOF. The final weight loss caused around from 800 °C to 1100 °C, along with an endothermic peak around 946.8 °C, which may be ascribed to the generation of Gd_2_O_3_ impurity phase. The results show three phase formation and the phase transformation among them, which agree well with the above-mentioned results from XRD patterns in Fig. 2(a).

The SEM images of 0.05 mol Er^3+^ doped GdOF precursor sintered at specified temperatures (0 °C, 650 °C, 800 °C, 1000 °C) for 3 h are shown in Fig. 1(e–h). Compared with the rice-shaped precursor in Fig. 1(e), the rice-shaped nanocrystals sintered at 650 °C in Fig. 1(f) maintain a monodisperse morphology with a smaller size due to getting rid of the surface organics as mentioned before. When further raised the sintered temperature to 800 °C and 1000 °C, the samples melted into some irregular pillars as shown in Fig. 1(g,h). The Er^3+^ doped GdOF nanorices tend to melt easier compared with the previous study on GdOF matrix^24^. One possible reason for this phenomenon is that the Er^3+^ ions enter into the crystal lattices of GdOF nanocrystals and replace the site of Gd^3+^, forming a solid solution at lower melting temperature.

**Figure 3:**
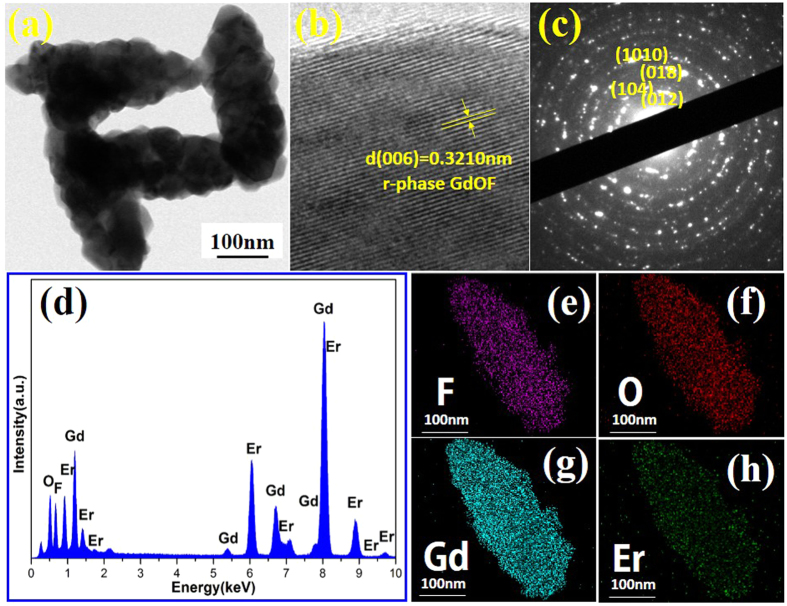
(**a–d**) TEM image, HRTEM image, SAED pattern and EDS spectrum of 0.05 mol Er^3+^ doped GdOF nanocrystals sintered at 650 °C, respectively. (**e–h**) The two-dimensional mapping distribution of F, O, Gd and Er, respectively.

Moreover, the morphology, composition and crystal structure of GdOF:0.05Er nanocrystals, which was heat-treated at 650 °C, were further characterized by TEM and HRTEM. Fig. 3(a) exhibits the TEM image of the product. These nanocrystals show the rice-shaped with width around 120 nm and length about 300 nm. Meanwhile, the corresponding HRTEM image of product (Fig. 3(b)) reveals that, the clear crystal lattice fringes with the spacing *d* value of 0.3164 nm corresponds to a (012) d-spacing of the rhombohedral phase GdOF:0.05Er (r-phase GdOF), which indicates GdOF rhombohedral phase exists in the product. Fig. 3(c) shows the electron diffraction (SAED) pattern of a selected area of the single GdOF:0.05Er nanocrystal. These clear diffraction rings imply that the sample displays a polycrystalline character. Moreover, EDS spectrum of corresponding product clearly shows the peaks according to the designed elements (Gd, O, F and Er) in Fig. 3(d). Furthermore, Fig. 3(e–h) are the two-dimensional mapping distribution of Gd, O, F and Er images which show the distribution region of Gd, O, F, and Er are almost uniform distribution in a single GdOF:0.05Er nano-rice. Therefore, it can be deduced from the results in Fig. 3(e–h) that the sample is mainly consisted of Gd, O, F and Er elements, and Er is evenly distributed in the GdOF matrix nanocrystals, which was heat-treated at 650 °C.

**Figure 4:**
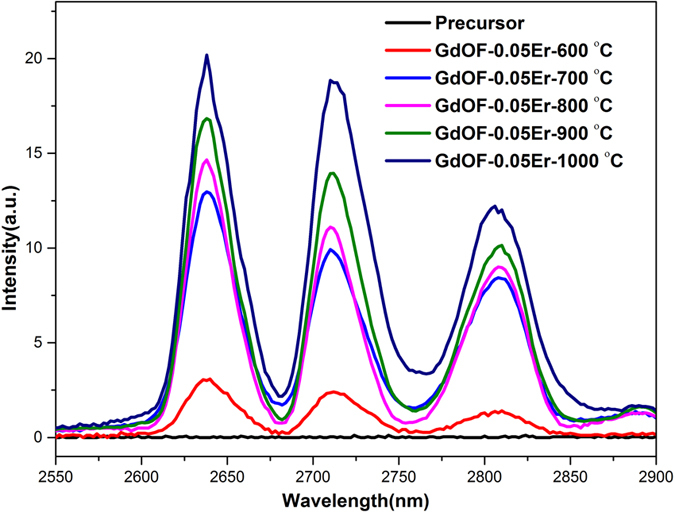
The 2.7 μm emission of 0.05 mol Er^3+^ doped GdOF precursor sintered at different temperatures for 3 h.

Herein, the mid-infrared emissions spectra (pumping by a 980 nm LD) of 0.05 mol Er^3+^ doped GdOF precursor sintered at different temperatures are displayed in Fig. 4. For the heat-treated GdOF samples, the broadband emissions from 2600 nm to 2900 nm, which correspond to Er^3+^: ^4^*I*_11/2_ → ^4^*I*_13/2_ transition, split into three peaks due to the Stark levels split^27^. In contrast, the unheated GdOF precursor is unable to give out any emission. With a sintering temperature at 600 °C, the 2.7 μm emission of Er^3+^ has realized in the Gd_4_O_3_F_6_ rice-like nanocrystals sample. In addition, an intense 2.7 μm emission of Er^3+^ has achieved in the GdOF nanocrystals at 700 °C, for the phase transition from Gd_4_O_3_F_6_ to GdOF at higher temperature. What is more, the intensity of 2.7 μm emission enhances with the calcination temperature increases, due to the better crystallinity and lower OH^−^ content in the products calcined at higher temperature.

### Controllable synthesis and mid-infrared properties of Er^3+^-doped YOF nanocrystals

In order to compare with the properties of GdOF:0.05Er, Er^3+^-doped YOF precursor was also prepared through a similar water-bath method in our experiments. The experiment parameter details of different precursor corresponding to YOF:0.05Er are presented in Supplementary Table 1. Supplementary Fig. S2(a–d) display the SEM images of 0.05 mol Er^3+^-doped YOF precursor samples. Overall, the samples present a morphology of nano-sphere shape. However, the size and distribution among these spherical precursors are different that can be observed in Supplementary Fig. S2(a–d). As the addition of 50 mmol urea, Supplementary Fig. S2(a) shows a uniform distribution of small nanospheres about 120 nm and large balls around 350 nm in the samples with 1 mmol KF in solution. When an equivalent solid KF is added, the smaller spheres still maintain a size around 100 nm, while the bigger ones are fewer in Supplementary Fig. S2(b). With an introduction of 1 mmol solid KF, as the amount of urea increase to 60 mmol, Supplementary Fig. S2(c) presents the YOF:0.05Er precursors in an average diameter of 100 nm with a good monodispersity. In addition, with 1.2 mmol solid KF and 60 mmol urea are added as a contrast, the quasi-spheres seem to be smaller than the above one, but slightly nonuniform in Supplementary Fig. S2(d). From the above results, it can be found that both the dosage of urea and the state of KF have much influence on the size distribution of the YOF:0.05Er precursor nanospheres, but less impact on the shape of products compared with that of GdOF:0.05Er. Therefore, the YOF**-**Er-c sample has been chosed as an optimum precursor to be investigated subsequently.

**Figure 5:**
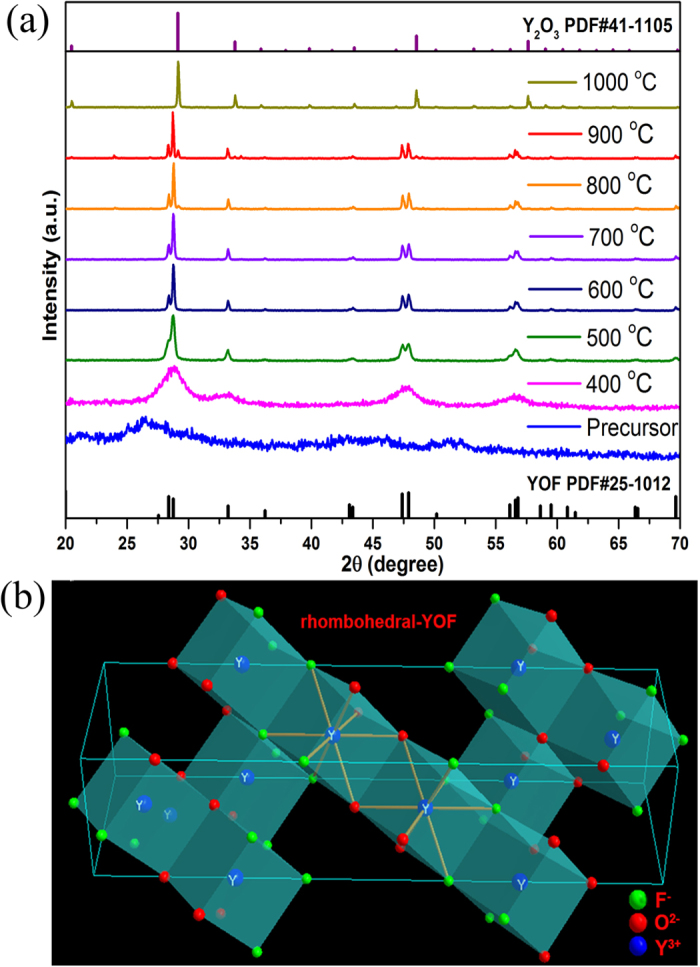
(**a**) XRD patterns of 0.05 mol Er^3+^ doped YOF precursor sintered at different temperatures for 3 h. (**b**) Crystal structure of rhombohedral YOF.

Fig. 5(a) shows the XRD patterns of as-prepared 0.05 mol Er^3+^-doped YOF precursor sintered at different temperatures (0 °C, 400 °C, 600 °C, 700 °C, 800 °C, 1000 °C). As shown in Fig. 5(a), the diffraction peaks in the XRD pattern of the amorphous precursor sample are not obvious. The broad diffraction peaks appeared when temperature reached 400 °C and these peaks can be assigned to the pure rhombohedral phase of YOF (a = b = 3.797 Å, c = 18.890 Å). When temperature reached 500–700 °C, the products still remained a pure rhombohedral YOF. The schematic view of the crystal structure of the rhombohedral YOF [space group: R-3m (166)] is also shown in Fig. 5(b). The Y^3+^ ions occupy sites with C_3v_ symmetry which are coordinated by four O^2−^ and four F^−^ ions, which is similar to the crystal structure of GdOF in Fig. 2(b). However, with further rising temperature, it can be noticed that a few diffraction peaks corresponding to cubic phase of Y_2_O_3_ (a = b = c = 10.604 Å) in the samples sintered 800 °C and 900 °C, but the main phase is still rhombohedral YOF. When the temperature reaches 1000 °C, the products present the pure cubic-Y_2_O_3_ for the oxygen atoms entering into crystals lattice replace all fluorine atoms. In addition, the corresponding TG-DSC curve of aforementioned precursor sample in Supplementary Fig. S3 exhibits obvious process weight loss. The endothermic peaks around 407.5 °C with a great mass loss from 280 °C to 500 °C, it might be due to the decomposition of the precursors and formation of the YOF crystalline phase. Another weight loss caused around from 860 °C to 1100 °C, along with an endothermic peak around 1016.8 °C, which may be ascribed to the generation of Y_2_O_3_ crystalline phase. The results agree well with the above-mentioned result analysis from XRD patterns in Fig. 5(a).

**Figure 6:**
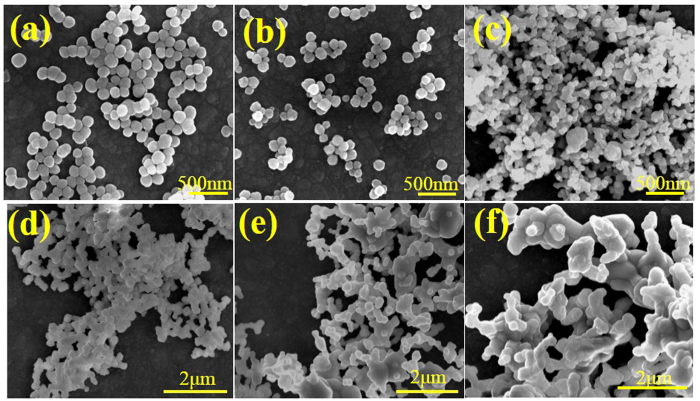
SEM images of 0.05 mol Er^3+^ doped YOF precursor sintered at different temperatures for 3 h: (**a**) 0 °C, (**b**) 400 °C, (**c**) 600 °C, (**d**) 700 °C, (**e**) 800 °C, (**f**) 1000 °C.

Furthermore, the SEM images of 0.05 mol Er^3+^-doped YOF precursor sintered at different temperatures (0 °C, 400 °C, 600 °C, 700 °C, 800 °C, 1000 °C) for 3 h are displayed in Fig. 6(a–f). The sample sintered at 400 °C (Fig. 6(b)) exhibits a morphology of monodisperse nano-sphere with a size around 80 nm, which is smaller than that of the as-prepared nano-spheres precursor in Fig. 6(a), due to the loss of surface organics. Fig. 6(c) shows the morphology of the samples sintered at 600 °C are well-dispersed grains with a size around 80 nm, but a slightly melting surface. When the sintered temperature increased to 700 °C, the sample (Fig. 6(d)) presents nano-spheres with an average diameter around 200 nm but a rough surface. Further elevating the sintered temperature to 800 °C, Fig. 6(e) shows the products presenting a morphology of nano-sphere crystals with more melt. In addition, it is remarkable that a widespread melting and interfacial boundary diffusion occur in the sample in Fig. 6(f). As the sintered temperature rises to 1000 °C, the nano-spheres are melting into a submicron bulk due to the completely phase transition from YOF to Y_2_O_3_.

**Figure 7:**
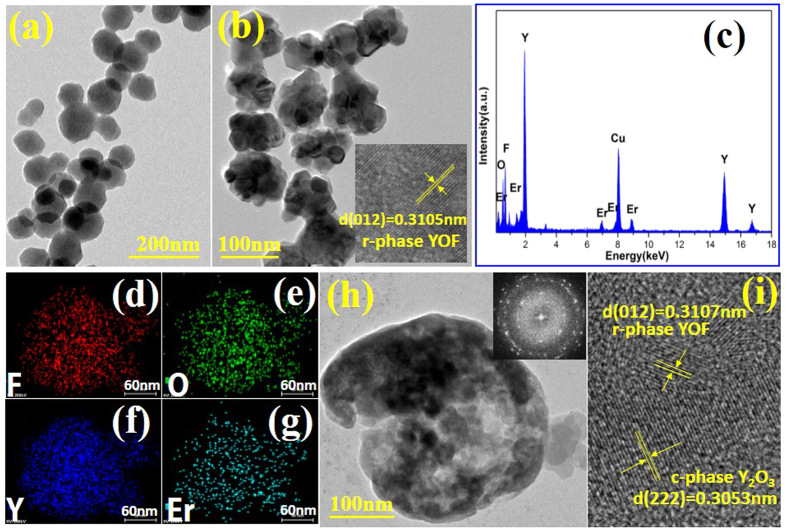
(**a**) TEM image of as-prepared 0.05 mol Er^3+^ doped precursor. (**b**) TEM image of as-prepared 0.05 mol Er^3+^ doped YOF nanocrystals sintered at 500 °C. The insert shows HRTEM image of single YOF:0.05Er nanocrystal sintered at 500 °C. (**c**) EDS spectrum of 0.05 mol Er^3+^ doped YOF nanocrystals sintered at 500 °C. (**d–g**) The two-dimensional mapping distribution of F, O, Y and Er in single Er^3+^ doped YOF nanocrystal, respectively. (**h**) TEM image of 0.05 mol Er^3+^ doped YOF nanocrystals sintered at 900 °C. The insert shows SAED pattern of single YOF:0.05Er nanocrystals sintered at 900 °C. (**i**) HRTEM image of 0.05 mol Er^3+^ doped YOF nanocrystals sintered at 900 °C.

The morphology, size and crystal structure of as-prepared precursor and YOF:0.05Er nanocrystals were further characterized by TEM and HRTEM. A typical TEM image of the precursor, Fig. 7(a) shows the nano-spheres with diameter around 80~100 nm. Fig. 7(b) is the TEM image of YOF:0.05Er nanocrystals sintered at 500 °C and shows the nano-spheres are structured by several smaller nanoparticles. The insert of Fig. 7(b) shows the corresponding HRTEM image which displays that the clear crystal lattice fringes with the spacing *d* value of 0.3105 nm corresponds to a (012) d-spacing of the rhombohedral phase YOF:0.05Er. And from Fig. 7(c) the peaks according to the designed elements (Y, O, F and Er) are both clearly observed in the corresponding EDS spectrum. In addition, the peaks of Cu originate from the TEM grid. Furthermore, the two-dimensional mapping distribution of Y, O, F and Er images are shown in Fig. 7(d–g) and illustrate that the distribution of Y, O, F, and Er are almost evenly. Thus, the results shown in Fig. 7(d–g) suggest that the sample is mainly consisted of Y, O, F and Er elements, and Er is uniformly distributed in the YOF matrix nanocrystals sintered at 500 °C. In addition, the TEM image and HRTEM image of 0.05 mol Er^3+^ doped YOF nanocrystals sintered at 900 °C are also displayed in Fig. 7(h,i). The sample (Fig. 7(h)) shows a nanosphere with several hundred nanometers and a melting surface. The insert of Fig. 7(h) shows the SAED pattern of a single YOF:0.05Er nanocrystal sintered at 900 °C is conducted by Fast Fourier Transform (FFT). The diffraction rings imply that the YOF:0.05Er nanocrystal exhibits a polycrystalline character. The corresponding HRTEM image of YOF:0.05Er nanocrystal sintered at 900 °C shown in Fig. 7(i). The crystal lattice fringes with different spacing *d* values of 0.3101 nm and 0.3065 nm can be measured directly, which probably correspond to the (012) crystal facets of the r-phase YOF and (222) crystal facets of the cubic phase Y_2_O_3_, respectively. Therefore, these results indicate the product calcined at 900 °C is consists of r-phase YOF and cubic phase Y_2_O_3_, which are in accordance with the XRD results in Fig. 5(a).

**Figure 8:**
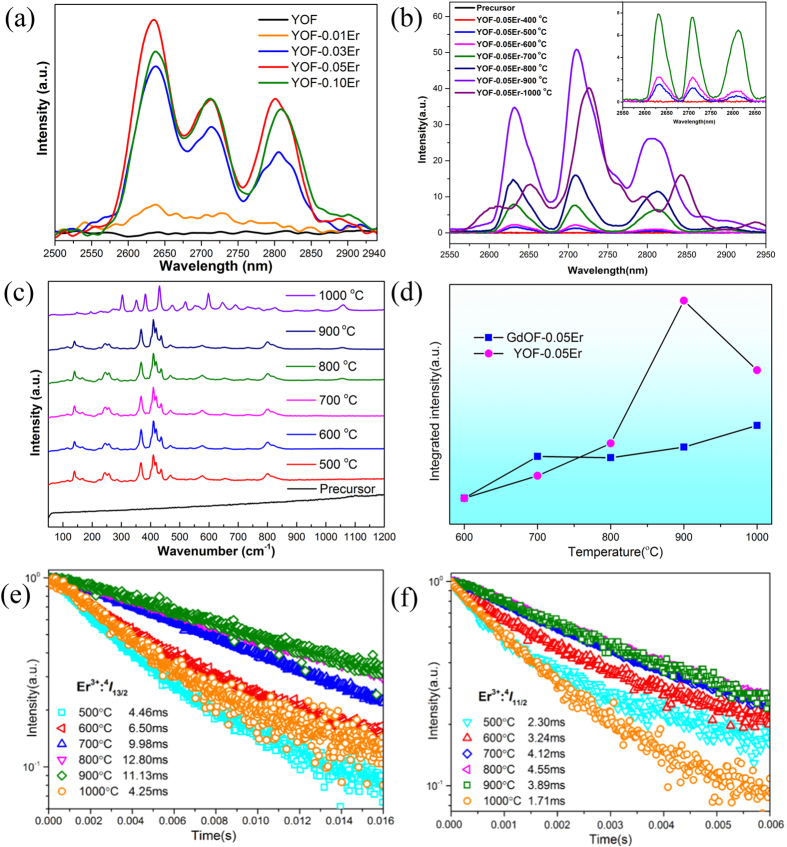
(**a**) The 2.7 μm emission of *x*Er^3+^ (*x* = 0, 0.01, 0.03, 0.05, 0.10 mol) doped YOF nanocrystals sintered at 600 °C for 3 h. (**b**) The 2.7 μm emission of 0.05 mol Er^3+^ doped YOF nanocrystals sintered at different temperatures for 3 h. (**c**) Raman spectra of undoped YOF precursor sintered at different temperatures for 3 h. (**d**) The integrated IR emission intensity from 2550 to 2900 nm of GdOF:0.05Er^3+^ and YOF:0.05Er^3+^ versus the calcination temperatures. (**e**,**f**) The fluorescence decay curves of the Er^3+^: ^4^*I*_11/2_ level (1007 nm) and ^4^*I*_13/2_ level (1530 nm) corresponding to 0.05 mol Er^3+^ doped YOF nanocrystals sintered at different temperatures for 3 h, respectively.

Fig. 8(a) presents the mid-infrared emissions spectra (under 980 nm LD pumping) of as-formed YOF:*x*Er (x = 0, 0.01, 0.03, 0.05, 0.10) nano-spheres sintered at 600 °C for 3 h. Compared to the spectrum of undoped YOF, emissions of Er^3+^: ^4^*I*_11/2_ → ^4^*I*_13/2_ transition that centred at 2.7 μm are clearly seen in other spectra. Moreover, the 2.7 μm emission intensity shows a tendency of increasing firstly then decreasing when the Er^3+^ doping concentration rises from 0.01 to 0.10. In order to obtained strongest 2.7 μm fluorescence, the optimum doping ratio of Er^3+^ ions is 0.05 as shown in these spectra. Since 0.05 mol Er^3+^ doped YOF gave optimum emission, we further studied the temperature effect on this type of samples. Fig. 8(b) shows the 2.7 μm Er^3+^ emissions of YOF:Er^3+^ precursors sintered at different temperatures for 3 h. Moreover, the intensity of 2.7 μm Er^3+^ emission tends to increase with an increasing sintering temperature from 500 to 900 °C. Especially, the spectrum of the sample sintered at 900 °C appears a great intensity enhancement. The results above may be ascribed to the following reasons: the crystallinity of samples becomes better at high temperature which is benefit for Er^3+^ ions entering into the crystalline lattice and giving out emissions. On the other hand, the samples tend to have larger size at higher temperature and may own fewer surface defects compared to the initial nanocrystals. Moreover, the other main factor of intensity enhancement may be hydroxyls are less in the structure of crystal particles if the calcination temperature is high^26^. It is worth noting that an obvious 2.7 μm mid-infrared emission is detected in YOF:0.05Er nanocrystals for the first time combined with the SEM and TEM results in Fig. 6 and 7. However, it can be also found that the sample heated at 1000 °C shows a noticeable decline in the 2.7 μm emission intensity, moreover, the broad emission splits into more peaks in the spectra. That may be due to the YOF phase completely transforms into Y_2_O_3_ crystalline as proposed in the XRD pattern (Fig. 5(a)), which leading to a change of crystal field. In addition, the Raman spectra of undoped YOF precursor sintered at different temperatures are shown in Fig. 8(c). From the pattern in Fig. 8(c), the phonon modes basically do not change with the calcination temperature from 500–900 °C, and the maximum phonon energy is about 800 cm^−1^. However, when the temperature rises to 1000 °C, the samples (Y_2_O_3_) exhibit a much wider distribution of phonon energies and the maximum phonon energy also increases to about 1050 cm^−1^. Namely the Y_2_O_3_ sample owns a higher phonon energy than YOF matrix, which exerts a detrimental influence to the 2.7 μm emission of Er^3+^ ions even though Y_2_O_3_ sample sintered at 1000 °C owns a much larger size.

## Discussion

Obviously, intense 2.7 μm emission of Er^3+^ ions has realized in both GdOF and YOF nanoparticles. In order to make a more intuitive comparisons, Fig. 8(d) directly displays the integrated IR emission intensity from 2550 to 2900 nm of GdOF:0.05Er and YOF:0.05Er versus the calcination temperature, respectively. It can be noted in the figure that the trend of the curve pertain to GdOF:Er^3+^ increases obviously and then flattens out with a rise of calcination temperature. However, in YOF matrix, the integrated emission intensity of Er^3+^ embarks on a steep rising trend firstly, and a sharp decline at 1000 °C just as suggested before. What is more, the emission intensity of Er^3+^ in GdOF host is slightly stronger than that of YOF host at a lower temperature at range from 600–700 °C. That may be because YOF:0.05Er nanocrystals possess a much smaller particle size than GdOF:Er^3+^ products calcined at 600–700 °C, resulting a higher density of surface defects, which impose a negative effect on Er^3+^:2.7 μm emissions. However, the emission intensity of the YOF:0.05Er crystals is more intense than that of GdOF:0.05Er at a higher temperature from 800 to 1000 °C, especially at 900 °C. The reason might be ascribed to the following: as suggested above, YOF:0.05Er crystals are equipped with better crystallinity, much larger size (from nanoscale to micron level) and fewer surface defects at high temperature, thus leading to an effective and intense Er^3+^:2.7 μm emissions. On the other hand, even though YOF host is similar to GdOF host in the structure, and both of them are rhombohedral phase, there are still some differences between them: Gd^3+^ ions (r = 0.938 Å, CN = 8) while Y^3+^ ions (r = 0.88 Å, CN = 8). When the Er^3+^ ions (r = 0.881 Å, CN = 8) are introduced to replace the Y^3+^ sites in the YOF crystal lattice, and the ionic radii is well matching with the Y^3+^ ions, which may be benefit to provide a stable lattice structure for Er^3+^ ions emitting intense 2.7 μm fluorescence. While, the mismatch between Er^3+^ and Gd^3+^ ions may result in a lattice distortion which might cause a unfavorable effect on the Er^3+^:2.7 μm emissions.

Furthermore, since the 2.7 μm emission of Er^3+^ is ascribed to the ^4^*I*_11/2_ → ^4^*I*_13/2_ transition, the fluorescence decay curves of the ^4^*I*_13/2_ (1530 nm emission) and ^4^*I*_11/2_ (1007 nm emission) levels in YOF hosts were measured and are shown in Fig. 8(e,f). The decay curves can be rigorously fitted to a single-exponential decay as shown in Fig. 8(e,f). It can be calculated that the fluorescence lifetime of ^4^*I*_11/2_ level presents a slightly extension from 2.30 ms to 4.55 ms, and subsequent shortening to 1.71 ms with an increase of calcination temperature. In addition, the ^4^*I*_13/2_ level presents a similar variation trend with a faster enhancement from 4.46 ms to 12.80 ms, and then decreasing to 4.25 ms.

Therefore, both rice-shaped GdOF:0.05Er nanocrystals and nano-sphere YOF:0.05Er can give out an intense 2.7 μm emission. And at lower calcination temperature, GdOF:0.05Er nanocrystals have a better performance in 2.7 μm fluorescence than YOF:0.05Er nanocrystals, whereas, Er^3+^ exhibits much more intense 2.7 μm emission in the YOF host than in the GdOF host at a high calcination temperature.

In conclusion, we have successfully synthesized the rice-like GdOF:Er^3+^ and nano-sphere YOF:Er^3+^ with mid-infrared emissions via a facile co-precipitation route and the subsequent heat-treatment. The state and dosage of KF and urea have great influences on the morphology and distribution of the precursor. Additionally, the phase transition happened from GdOF (YOF) nanocrystals to their corresponding oxide Gd_2_O_3_ (Y_2_O_3_) as increasing the heat-treatment temperature, meanwhile the morphologies of GdOF: Er^3+^ and YOF: Er^3+^ nanocrystals also changed. The mid-infrared fluorescence spectra suggest that 2.7 μm emissions originated from Er^3+^ ions have achieved in both GdOF and YOF nanocrystals, and the emission intensity tends to increase with the calcination temperature. Moreover, the fluorescence decay curves of the ^4^*I*_13/2_ and ^4^*I*_11/2_ levels in YOF hosts were also measured. In this paper, both GdOF nanocrystals and YOF nanocrystals with mid-infrared emissions are prepared and they could be suitable candidate materials as luminescence center for nanocrystals-glass composites, which would pave a way to the realization of novel mid-infrared fluorescence and laser materials.

## Methods

### Preparation of LnOF:xEr (Ln = Gd, Y) nanocrystals

LnOF:*x*Er (Ln = Gd, Y) nanocrystals were synthesized via a co-precipitation route and subsequent heat-treatment^28^,^29^. Take GdOF:*x*Er as an example, 1 mmol stoichiometric of mixed Gd(NO_3_)_3_ and Er(NO_3_)_3_ were added into calculated deionized water to form a 100 mL of aqueous solution finally. With a vigorous magnetic stirring, 1 mmol KF and a certain quantity of CO(NH_2_)_2_ were added to the solution, respectively. Dilute HNO_3_ was dropped rapidly into the vigorously stirred solution until pH = 2 (pH = 4 in YOF). After magnetic stirring for another 30 min, the mixed solution was immersed in a constant temperature bath with stirring and maintained at 85 °C for 3 h. The resulted precipitate was cooled to room temperature, and collected by centrifugation with a rotator speed of 10000 rpm for 5 min. The collection was washed several times with deionized water and ethanol successively, then dried at 80 °C for 24 h. Afterwards, a heat-treatment (temperature continue rising with a rate of 1 °C/min for 3 h) was applied on the precursor which was contained in an alumina crucible, then the GdOF:*x*Er nanocrystal samples were obtained. The doped YOF samples were prepared by introducing the proper amounts of Y(NO_3_)_3_ instead of Gd(NO_3_)_3_ to the solution as described above.

### Characterization

The crystalline structures of nanocrystals were characterized by X-ray diffraction (XRD) in a D8 advance X-ray diffractometer (Bruker, Switzerland) at a scanning rate of 0.2 °/min with a Cu Kα radiation (151.54056 A°). The morphology, size distribution and composition of as-prepared nanocrystals were investigated by the field emission-scanning electron microscopy (FE-SEM, Nova NanoSEM430, FEI, Netherlands) with an energy-dispersive X-ray spectrometer (EDS) and high-resolution transmission electron microscopy (HR-TEM, 2100F, JEOL, Japan). Thermal analysis of the precursor samples were studied by using a simultaneous thermal analyzer (STA, STA449C/3/MFC/GJUPITEY, NETZSCH, Germany) with a heating rate of 10 °C/min. Raman spectra were tested in a Renishaw inva spectrometer with a 532 nm laser as the pump source. Photoluminescence spectra were investigated using a liquid nitrogen refrigeration type DInSb5-De02 infrared detector, cooperating with DCS300PA data collector and the phase-locked amplifier for data acquisition. Fluorescence lifetimes were collected with a iHR 320 spectrometer equipped with a TDS 3012B digital oscilloscope (Tektronix, USA), associated with a 808 nm or a 980 nm pulse LD laser (LEO Photoelectric, China), respectively. All the measurements were conducted at room temperature.

## Additional Information

**How to cite this article**: He, H. *et al.* Controllable Synthesis of Monodisperse Er^3+^-Doped Lanthanide Oxyfluorides Nanocrystals with Intense Mid-Infrared Emission. *Sci. Rep.* **6**, 35348; doi: 10.1038/srep35348 (2016).

## Supplementary Material

Supplementary Information
