# Supplementary material for: Controllable Synthesis of Monodisperse Er3+-Doped Lanthanide Oxyfluorides Nanocrystals with Intense Mid-Infrared Emission
Source: Sci Rep. 2016 Oct 17;6:35348. doi: 10.1038/srep35348 (PMC5066252; doi:10.1038/srep35348)
Supplement: Supplementary Information [file srep35348-s1.pdf]

Supporting information

Controllable Synthesis of Monodisperse Er^{3+}-Doped Lanthanide Oxyfluorides Nanocrystals with Intense Mid-Infrared Emission

Huilin He, Qiang Liu, Dandan Yang, Qiwen Pan, Jianrong Qiu, Guoping Dong*

State Key Laboratory of Luminescent Materials and Devices and Guangdong Provincial Key Laboratory of Fiber Laser Materials and Applied Techniques, School of Materials Science and Engineering, South China University of Technology, Guangzhou 510640, China

*Correspondence to [dgp@scut.edu.cn (G. Dong)]

Precursor	$[Urea^{3+}]/[Ln^{3+}]$	KF		pH	Water bath Temperature (°C)	Water bath Time (h)
		Solid (g)	Solution (mL) (0.4mol/L)			
GdOF-Er-a	33mmol	-	2.5 (1mmol)	2	85	3
GdOF-Er-b	33mmol	0.0581 (1mmol)	-	2	85	3
GdOF-Er-c	33mmol	-	3 (1.2mmol)	2	85	3
GdOF-Er-d	33mmol	0.0697 (1.2mmol)	-	2	85	3
YOF-Er-a	50mmol	-	2.5 (1mmol)	4	85	3
YOF-Er-b	50mmol	0.0581 (1mmol)	-	4	85	3
YOF-Er-c	60mmol	0.0581 (1mmol)	-	4	85	3
YOF-Er-d	60mmol	0.0697 (1.2mmol)	-	4	85	3

Table S1 Experiment parameters of different precursors corresponding to GdOF:0.05Er and YOF:0.05Er.

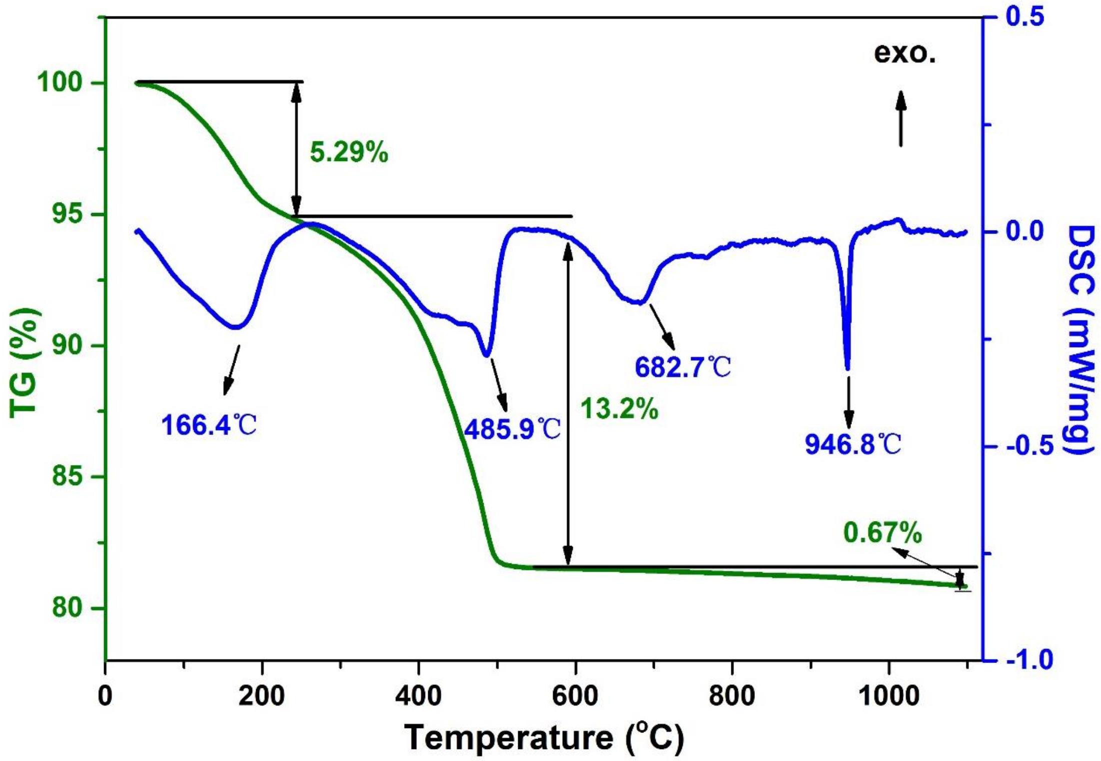

Figure S1 TG-DSC curves of the 0.05 mol Er^{3+} doped GdOF precursor in air atmosphere with a heating rate of 10 °C/min.

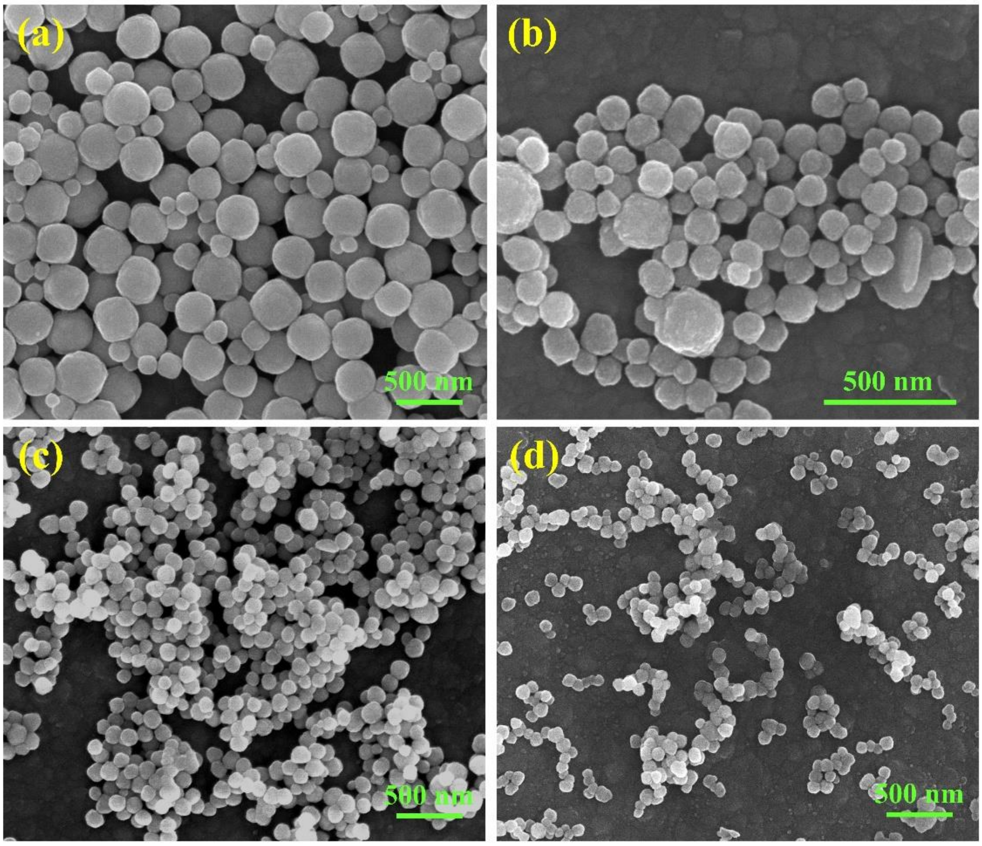

Figure S2 (a-d) SEM images of 0.05 mol Er^{3+} doped YOF precursor: pH = 4, (a) YOF-Er-a (1 mmol KF solution, 50 mmol urea), (b) YOF-Er-b (1 mmol KF solid powder, 50 mmol urea), (c) YOF-Er-c (1 mmol KF solid powder, 60 mmol urea), (d) YOF-Er-d (1.2 mmol KF solid powder, 60 mmol urea).

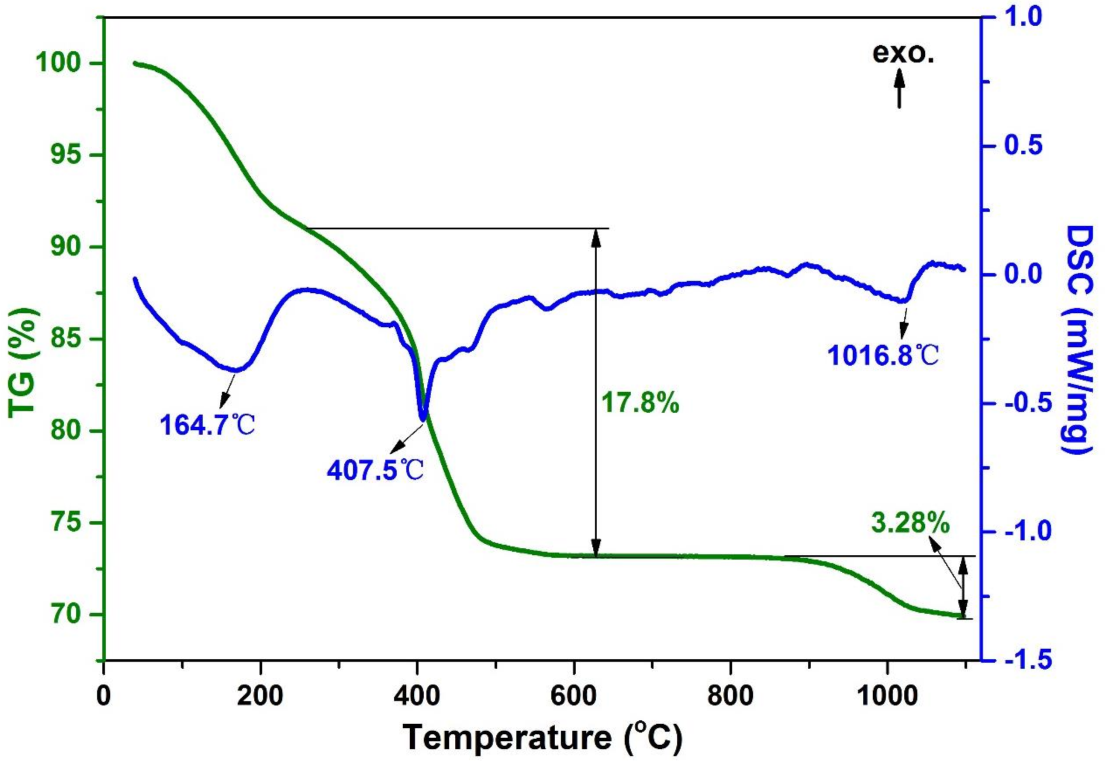

Figure S3 TG-DSC curves of the 0.05 mol Er^{3+} doped YOF precursor in air atmosphere with a heating rate of 10 °C/min.
